# Treatment of a Rare Case of Orbital Necrotizing Fasciitis Utilizing Negative Pressure Wound Therapy

**DOI:** 10.7759/cureus.18682

**Published:** 2021-10-11

**Authors:** Akshay J Reddy, Nathaniel Tak, Neel Nawathey, Samuel A Habib, James B Martel

**Affiliations:** 1 Ophthalmology, California Northstate University College of Medicine, Elk Grove, USA; 2 Ophthalmology, Martel Eye Medical Group, Rancho Cordova, USA; 3 Health Sciences, California Northstate University, Rancho Cordova, USA; 4 Health Sciences, Santa Clara University, Santa Clara, USA

**Keywords:** edema, skin graft, periorbital cellulitis, necrotizing fasciitis, vacuum assisted closure (vac)

## Abstract

We present a severe case of orbital necrotizing fasciitis that was treated utilizing negative pressure wound therapy (NPWT).

The conditions caused by the disease and the utility of the treatment were discussed. Additionally, the functionality and the process of the treatment were thoroughly analyzed. Potential complications from utilizing NPWT were also identified. When the patient was tested, it was found that he had intra op cultures with group B Streptococcus *pyogenes *(Strep pyogenes). CT scans were also conducted to analyze his right lateral periorbital tissue. Subsequently, the patient was admitted to the ICU, where a wound vacuum-assisted closure (VAC) was placed on his right eye. Once the NPWT was complete, the patient was prescribed antibiotics and was able to improve the health within his right eye.

## Introduction

Although negative pressure wound therapy (NPWT) is primarily utilized to heal acute and chronic wounds, the treatment option can be applied to numerous other clinical problems [1]. This treatment option can help drain the tissue of fluids and pathogens that are causing problems within a patient’s body, help wounds heal at a faster rate, and even help with certain skin grafting procedures. Current estimates suggest that NPWT is used to help treat over 40% of surgical site infections [2]. Necrotizing fasciitis is an extremely rare and deadly disease that is caused by flesh-eating bacteria that penetrates the skin [3]. Within the United States, approximately 1000 people per year die from this disease [4]. This disease could lead to a variety of serious complications, including death if it is not treated early enough using the right procedures or equipment. Therefore, it is important that physicians are aware of the success rate of various procedures, such as NPWT, in order to ascertain the best course of treatment for their patients.

## Case presentation

A 44-year-old Caucasian man presented with worsening swelling, tenderness, and erythema around his right eye, including the right side of his face. According to the patient, he woke up in the morning with swelling of the right eye and did not think anything of it. He thought it would improve. However, in less than 24 hours, he had extensive right facial swelling extending all the way to the submandibular region. On presentation, apparently, he had extension across to the left periorbital region and the premaxillary region as well. He did not note any leukocytosis; however, had bandemia. He was noted to have elevated liver enzyme tests (LETs), also noted to be in alcohol withdrawal with severe shakiness. It was found through intraop cultures that he tested positive for having group B Streptococcus pyogenes (Strep pyogenes), which indicated that he had necrotizing fasciitis. Subsequently, he underwent debridement and placement of wound VAC to the right upper and lower eyelids. The treatment involved using debridement and wound VAC placement in order to treat his septic shock in the setting of severe right facial cellulitis along with right periorbital cellulitis. 

**Figure 1:**
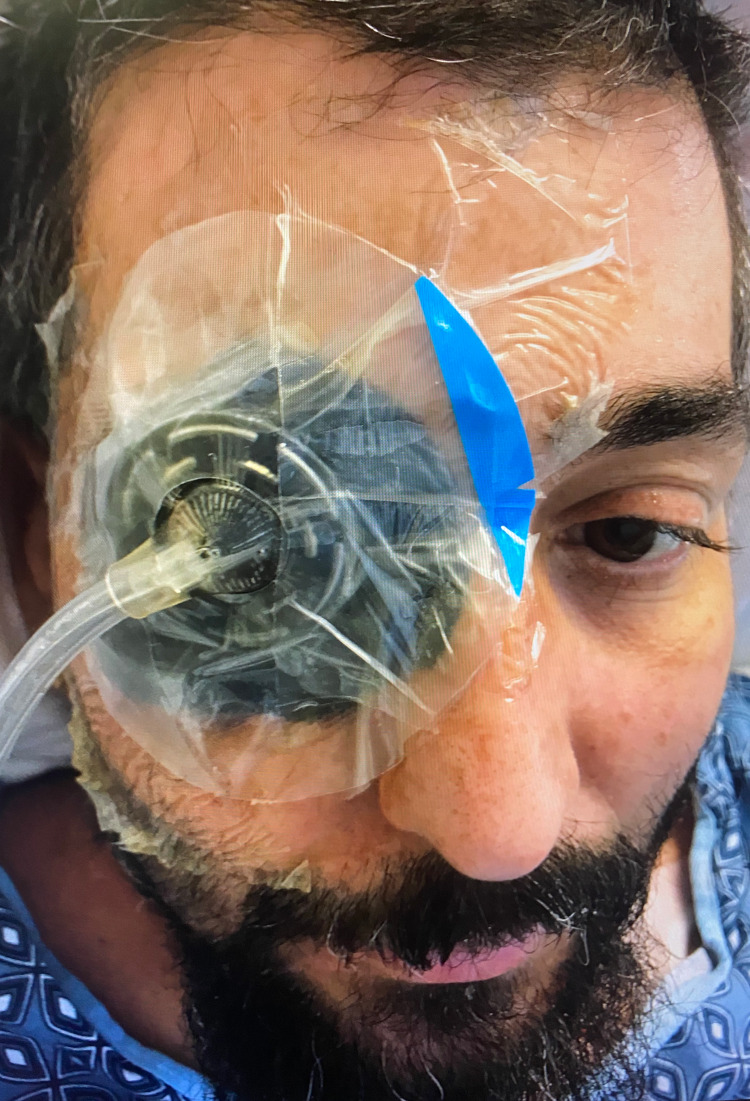
Using a wound VAC to implement NPWT on a patient with necrotizing fasciitis. VAC: Vacuum-assisted closure; NPWT: Negative pressure wound therapy.

The patient was brought to the operating room and placed in a supine position under general anesthesia. He had an inspection. There was a VAC that was present with some sort of adhesive toward the nose, but essentially was drying at an adequate pressure. The patient had been getting IV antibiotics and once the operative report was obtained, the procedure began with attention centered to the right upper eyelid, which looks exceedingly improved compared to its original condition. The nasal aspect of the wound showed no necrosis. There was granulation tissue formation. The temporal aspect shows some level of necrosis and this was removed down to a vital muscularis region. The inferior area of necrosis looked well as far as the midline to the canthus, however, the lateral aspect required quite a bit more dissection down through, but external to the orbicularis. A bleeder was noted as dissection was made down through the maxilla and was cauterized. The area was dry, so a moist 4 X 4 wet-to-dry gauze was placed and then a Tegaderm was placed over it. If the nursing staff had been here, the VAC could have been placed, however, given the circumstances, we placed the VAC in the ICU with the patient being given proper sedation (Figure 1). The patient tolerated the procedure well and went back into the ICU. 

Post-operation, he had interval improvement with his IV antibiotics. There was decreased swelling. His extraocular eye movements were full. There is a skin graft over the right upper eyelid, which extends through the areas of previous necrosis, and there is periorbital edema. He was able to open his eyes. There was no discharge in the conjunctiva. We recommended that the head of his bed should be elevated, as this will help decrease the edema around his left eye. The patient had pressures of 20 mmHg in both eyes. His cornea was clear and his anterior chamber was well-formed. His preseptal region was more edematous than previously reported two weeks ago when the sutures were removed. The patient was prescribed to have antibiotic bacitracin ointment once a day. The CT scan was noticeable for right lateral periorbital soft tissue swelling without any adjacent fracture. This was most likely the rotational flap. Otherwise, there was decreased swelling as compared with previous examinations. The maxillofacial CT scan was normal. He had interval improvement with his IV antibiotics.

## Discussion

Necrotizing fasciitis is an infection that primarily affects soft tissue. There are four major types of necrotizing fasciitis: types I through IV [5]. Type I is the most common and is caused by polymicrobial infection. Both types II and III are caused by mono-microbial infections. Type II is primarily caused by Strep pyogenes. Type III is typically caused by a clostridium bacterial species. Type IV is caused by a fungal infection [6]. Without proper and immediate treatment, the infection can spread rapidly causing cell and tissue death and complications such as sepsis, shock, and organ failure. The patient in this study had type II necrotizing fasciitis. 

The main goal when treating necrotizing fasciitis is to prevent its spread. Since necrotizing fasciitis can spread at an alarming rate, it is quintessential to ensure that patients get diagnosed as fast as possible. Typically, treatments for necrotizing fasciitis involve the use of antibiotics as well as surgery to quickly remove the infection. 

NPWT is a treatment method that involves removing the fluid from a wound in order to aid its healing process. This is done with a vacuum pump as well as a foam pad to preserve the pressure seal around the wound. The pressure seal also serves as a means to prevent the wound from further bacterial infections. This negative pressure creates a means to extract exudate from the wound tissue. This is material that has failed to be uptaken by the blood vessels and remains in the surrounding tissue. The exudate is then removed from the wound via the foam pad into a container where it can be disposed of [7]. 

There are several benefits in utilizing VAC such as increased growth rate and diminution of the wound area [8]. The presence of the foam pad along with the negative pressure contributes to the immobilization of the wound. This aids in the healing process. Due to the hypobaric interstitial pressure created from a wound, there is increased blood vessel permeability, allowing for the formation of edema [8]. A VAC combats this by creating compression within the tissues and vessels. The compression of the blood vessels increases the velocity of the intravascular fluid. This reduces the efflux of intravascular fluid within the wound and thus decreases edema. Instead, extracellular fluid is uptaken by the blood vessel. In doing so, there is increased oxygenation of cells in the affected area allowing for better tissue repair [9]. As result of the pressure differential within and outside of the tissues and increased internal cellular pressure, it causes the cells to expand, and the growth of granulation tissues aiding in the closure of the wound. This increased growth is aided by the micro deformation of cells caused by the negative pressure environment. 

This treatment is not without its dangers. If the vascular tree around the wound is normal, the pressure differential is unlikely to cause complications, such as capillary occlusion. However, if the patient is ischemic, the negative pressure may lead to ischemia and necrosis. Furthermore, complications can arise during VAC therapy, such as excess bleeding, excoriation of skin, adherence of tissue to the applied foam, and skin necrosis [10].

In this study, a wound VAC was placed on the right upper and lower eyelids. While the VAC could have been administered earlier, it was ultimately placed when the patient was in the ICU. Post-operation, there was an improvement to the affected area in combination with IV antibiotics. The patient had no discharge in the conjunctival, clear cornea, well-formed anterior chamber, full extraocular eye movements as well as the ability to open the eyelids.

## Conclusions

NPWT can be an effective treatment option when attempting to help reduce the pertinence of necrotizing fasciitis. Although this treatment can be effective, it is important to note that in order to apply the wound VAC, the right equipment and staff is necessary. In this study, the wound VAC could only be applied to the patient when brought to the ICU as there were not enough nurses on staff. The patient was able to recover functionality in his right eye after the full treatment was complete. Currently, there is no set standard for when physicians should use NPWT to treat necrotizing fasciitis, however, it can and is generally used as a method to treat severe cases of infection and swelling caused by necrotizing fasciitis.
